# Supplementary material for: Multiple channelling single-photon emission with scattering holography designed metasurfaces
Source: Nat Commun. 2023 Oct 6;14:6253. doi: 10.1038/s41467-023-42046-3 (PMC10558519; doi:10.1038/s41467-023-42046-3)
Supplement: Supplementary file 1 — Supplementary information [file 41467_2023_42046_MOESM1_ESM.pdf]

Supporting Information: Multiple channelling single-photon emission with scattering holography designed metasurfaces

Danylo Komisar,[*,†] Shailesh Kumar,[*,†] Yinhui Kan,[†] Chao Meng,[†] Liudmila F. Kulikova,[‡] Valery A. Davydov,[‡] Viatcheslav N. Agafonov,[¶] and Sergey I. Bozhevolnyi[†,§]

†*Center for Nano Optics, University of Southern Denmark, DK-5230 Odense M, Denmark*

‡*L.F. Vereshchagin Institute for High Pressure Physics, Russian Academy of Sciences, Troitsk, Moscow, 142190, Russia*

¶*GREMAN, UMR CNRS CEA 6157, Université F. Rabelais, 37200 Tours, France*

§*Danish Institute for Advanced Study, University of Southern Denmark, Campusvej 55, DK-5230 Odense M, Denmark*

E-mail: dak@mci.sdu.dk; shku@mci.sdu.dk

Contents

S1 Principles of scattering holography **S-3**

S1.1 Holographic approach: shaping QE emission S-3

S1.2 Holographic approach: channeling QE emission into two beams S-6

S1.3 Cylindrically diverging SPP as a reference wave S-8

S2 Calculation of metasurface pattern for reconstruction of two signal waves of orthogonal linear polarizations **S-9**

S2.1 Initial design S-13

S2.2 Optimized design S-15

S2.3 Performance comparison of initial and optimized metasurface design S-17

S2.4 Performance comparison of metasurfaces of different sizes S-19

S3 Quantum efficiency calculation **S-20**

S4 Metasurface function robustness against shift of the ND **S-25**

S5 Metasurface fabrication **S-26**

S6 Experimental setup **S-29**

S7 Experiment repeatability **S-31**

S7.1 Characterization of hologram metasurface coupled to another single-photon GeV S-31

S7.2 Characterization of hologram metasurface coupled to SiV ND S-33

Supplementary References **S-35**

S1 Principles of scattering holography

Holography is an approach used for recording and reconstructing arbitrary wavefronts. A hologram is produced by recording an interference pattern between a reference wave and a signal wave, typically by using a photosensitive thin film. When illuminated with the reference wave, the hologram, representing usually an amplitude or phase-encoded transparency, reproduces (with certain accuracy) the signal wave phase and amplitude distribution. The intensity interference pattern can also be calculated and thereby imprinted into a thin film (varying either its amplitude transmission or thickness), resulting in a computer-generated hologram. In this section, we describe our holographic method, scattering holography[S1], developed for designing metasurfaces that produce far-field beams of predefined directions and polarizations.

We discuss in the following the case of a cylindrically diverging SPP wave, which is assumed to be excited by a quantum emitter (QE), used as a reference wave interacting with a (computer-generated) metasurface and scattering into two orthogonally linearly polarized beams separated in their direction of propagation.

S1.1 Holographic approach: shaping QE emission

First, we present a general method of recording the holographic pattern and reconstruction of the signal wave using SPP as a reference wave. Then, we elaborate on the case of QE-excited cylindrically diverging SPP and provide corrections to the model.

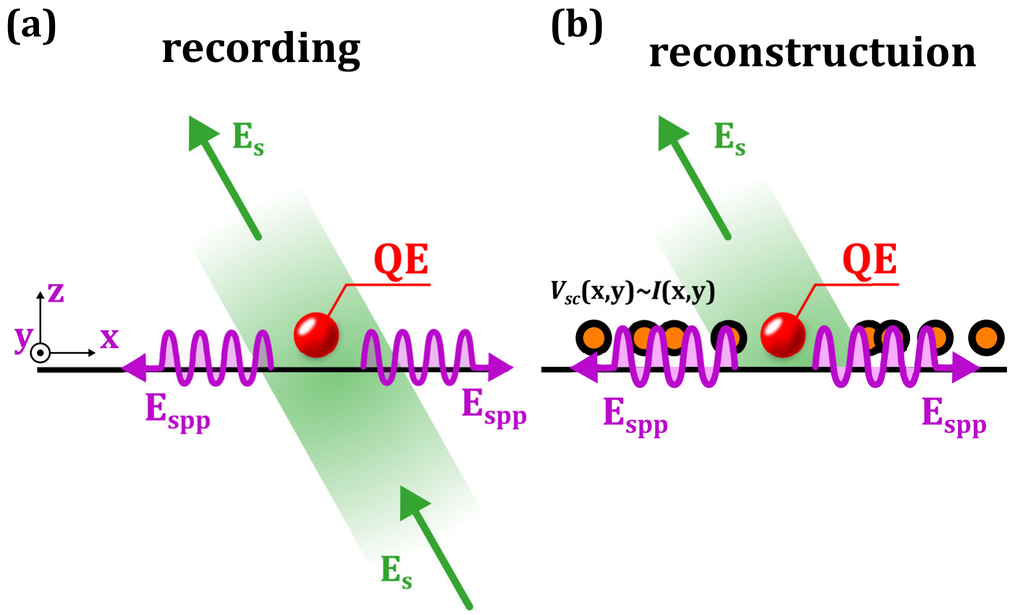

Figure S1: Concept of scattering holography with cylindrically diverging SPP generated by a quantum emitter (QE). Calculation of the interference pattern intensity generated by a signal wave $\mathbf{E_s}$ and SPP reference wave $\mathbf{E_{spp}}$ in the hologram xy-plane (a), and reconstruction of the signal wave by scattering of the reference SPP wave by nanoparticle distribution matching the interference pattern (b).

As a starting point, we consider the interference between the reference SPP wave $\mathbf{E_{spp}}$ and the signal wave $\mathbf{E_s}$ at **xy-plane** [z=0, Figure S1(a)]. The interference pattern can be written as:

$$I(x,y) = \left|\mathbf{E_{spp}} + \mathbf{E_s}\right|^2 = \left|\mathbf{E^0_{spp}} \exp(-i\mathbf{k_{spp}} \cdot \mathbf{r_{xy}}) + \mathbf{E^0_s} \exp(-i\mathbf{k_s} \cdot \mathbf{r_{xy}})\right|^2 =$$

$$= I^0_{spp} + I^0_s + \mathbf{E^0_{spp}} \cdot \mathbf{E^0_s}^* \exp[-i(\mathbf{k_{spp}} \cdot \mathbf{r_{xy}} - \mathbf{k_s} \cdot \mathbf{r_{xy}})] + \mathbf{E^0_{spp}}^* \cdot \mathbf{E^0_s} \exp[-i(\mathbf{k_s} \cdot \mathbf{r_{xy}} - \mathbf{k_{spp}} \cdot \mathbf{r_{xy}})] \quad \text{(S1)}$$

where $\mathbf{E^0_{spp}}$ and $\mathbf{E^0_s}$ denote the complex vectorial electric field amplitudes, while $\mathbf{k_s}$ and $\mathbf{k_{spp}}$ represent wavevectors for the signal and reference SPP waves, respectively. All quantities in equation S1 are **functions of coordinates** (x, y). The in-plane radius vector is denoted by $\mathbf{r_{xy}}$. Here, the in-plane electric foeld component of QE-excited SPP $\mathbf{E^0_{spp}}$ is oriented radially: $\mathbf{E^0_{spp}}(x,y) = \left|\mathbf{E^0_{spp}}(x,y)\right|(cos(\varphi), sin(\varphi))$, where φ is the angle between **x**-axis and the SPP propagation direction $\mathbf{k_{spp}}$. The in-plane components of vectors $\mathbf{k_{spp}}$ and $\mathbf{r_{xy}}$ are parallel to each other,

therefore $\mathbf{k_{spp}} \cdot \mathbf{r_{xy}} = k_{spp} r_{xy}$.

We consider the hologram to be composed of **isotropic, non-interacting dipolar nano-scatterers**, for example, **spherical** nanoparticles[S1]. The volumes of these nanoparticles, and hence their scalar polarizabilities, are considered to be proportional to the intensity of the interference pattern: $V_{sc}(x, y) \sim I(x, y)$. The size of scatterers is assumed to be much smaller than the operating light wavelength so that one could employ the electric-dipole approximation when considering radiation scattering by the hologram nanoparticles.

When such a hologram of distributed nanoparticles is illuminated with the reference SPP wave, the scattered field in the immediate vicinity of a nanoparticle is proportional to the incident field and the nanoparticle polarizability, which is proportional to the nanoparticle volume and thus to the local intensity of the interference pattern:

$$\begin{gathered} \mathbf{E_{sc}}(x, y) \sim I(x, y)\mathbf{E_{spp}} = (I_r^0 + I_s^0)\mathbf{E_{spp}}^0 \exp(-ik_{spp} \cdot r_{xy}) + \\ +(\mathbf{E_{spp}}^0 \cdot \mathbf{E_s^0}^*)\mathbf{E_{spp}}^0 \exp[-i(2k_{spp} r_{xy} - \mathbf{k_s} \cdot \mathbf{r_{xy}})] + \\ (\mathbf{E_{spp}^{0^*}} \cdot \mathbf{E_s^0})\mathbf{E_{spp}}^0 \exp[-i\mathbf{k_s} \cdot \mathbf{r_{xy}}] \end{gathered} \tag{S2}$$

The first term of the scattered field represents the evanescent field with $\mathbf{k_{spp}} > \mathbf{k_0}$ which doesn't propagate away from the hologram's **xy** plane. The second term of $\mathbf{E_{sc}}$ is also a characteristic evanescent wave because the associated wavevector magnitude is larger than wavenumber k_0 of a free propagating wave, $|2\mathbf{k_{spp}} - \mathbf{k_s}| > k_{spp} > k_0$. As a result, the only reconstructed wave, which propagates away from the hologram surface, is the reconstructed signal wave represented by the last term: $\mathbf{E_{sc}}$. This propagating away scattered field propagates in the direction of the signal wave [Figure S1(b)], but has the polarization of the reference wave:

$$\mathbf{E_{sc}} \sim I(x, y) \cdot \mathbf{E_{spp}} \sim (\mathbf{E_{spp}^{0^*}} \cdot \mathbf{E_s^0})\mathbf{E_{spp}^0} \exp[-i\mathbf{k_s} \cdot \mathbf{r_{xy}}] \tag{S3}$$

S1.2 Holographic approach: channeling QE emission into two beams

In this section, we consider the case of holographic reconstruction of two beams with orthogonal polarizations.

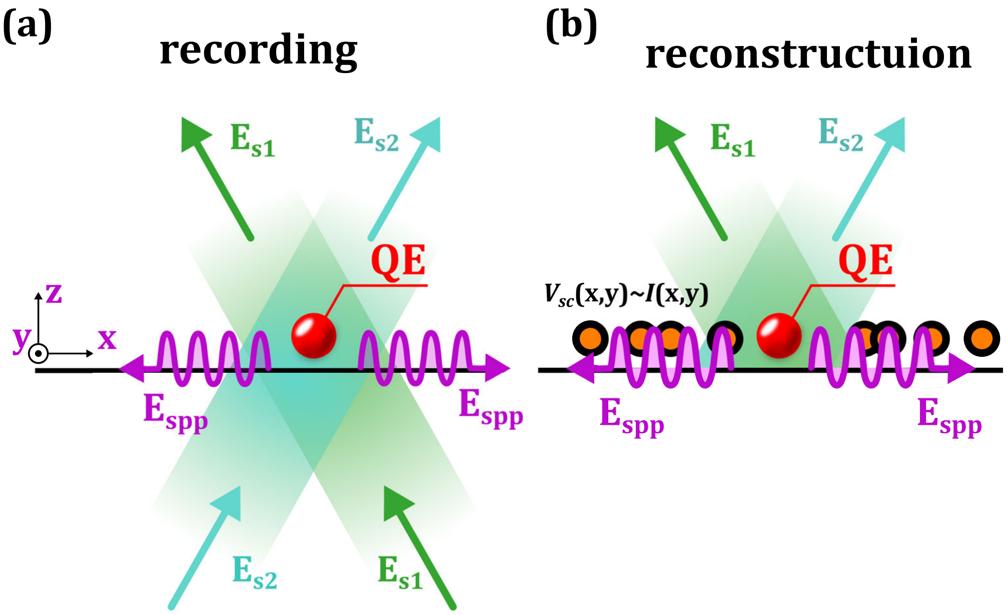

Figure S2: A schematic of vectorial scattering holography with cylindrically diverging SPP generated by quantum emitter (QE). Calculation of the interference pattern intensity generated by two signal waves $\mathbf{E_{s1}}, \mathbf{E_{s2}}$ and SPP reference wave $\mathbf{E_{spp}}$ in the hologram xy-plane (a), and reconstruction of the signal wave by scattering of the reference SPP wave by the nanoparticle distribution matching the interference pattern (b).

The interference between the reference $\mathbf{E_{spp}}$ and the two signal waves $\mathbf{E_{s1}}, \mathbf{E_{s2}}$ [Figure S2(a)] can be calculated as follows:

$$I(x,y) = \left|\mathbf{E_{spp}} + \mathbf{E_{s1}} + \mathbf{E_{s2}}\right|^2 =$$
$$= \left|\mathbf{E^0_{spp}} \exp(-ik_{spp}r_{xy}) + \mathbf{E^0_{s1}} \exp(-i\mathbf{k_{s1}}\cdot\mathbf{r_{xy}}) + \mathbf{E^0_{s2}} \exp(-i\mathbf{k_{s2}}\cdot\mathbf{r_{xy}})\right|^2 =$$
$$= I^0_{spp} + I^0_x + I^0_y +$$
$$+\mathbf{E^0_{spp}}\cdot{\mathbf{E^0_{s1}}}^* \exp[-i(k_{spp}r_{xy} - \mathbf{k_{s1}}\cdot\mathbf{r_{xy}})] + \mathbf{E^{0*}_{spp}}\cdot\mathbf{E^0_{s1}} \exp[-i(\mathbf{k_{s1}}\cdot\mathbf{r_{xy}} - k_{spp}r_{xy})]+ \tag{S4}$$
$$+\mathbf{E^0_{spp}}\cdot{\mathbf{E^0_{s2}}}^* \exp[-i(k_{spp}r_{xy} - \mathbf{k_{s2}}\cdot\mathbf{r_{xy}})] + \mathbf{E^{0*}_{spp}}\cdot\mathbf{E^0_{s2}} \exp[-i(\mathbf{k_{s2}}\cdot\mathbf{r_{xy}} - k_{spp}r_{xy})]+$$
$$+\mathbf{E^0_{s1}}\cdot{\mathbf{E^0_{s2}}}^* \exp[-i(\mathbf{k_{s1}}\cdot\mathbf{r_{xy}} - \mathbf{k_{s2}}\cdot\mathbf{r_{xy}})] + {\mathbf{E^0_{s1}}}^*\cdot\mathbf{E^0_{s2}} \exp[-i(\mathbf{k_{s2}}\cdot\mathbf{r_{xy}} - \mathbf{k_{s1}}\cdot\mathbf{r_{xy}})]$$

The field reconstructed by the holographic array of nano-scatterers can then be written down:

$$\mathbf{E_{sc}}(\mathbf{x},\mathbf{y}) = I(x,y)\mathbf{E_{spp}} = (I^0_{spp} + I^0_x + I^0_y)\mathbf{E^0_{spp}} \exp(-ik_{spp}r_{xy})+$$
$$+(\mathbf{E^0_{spp}}\cdot{\mathbf{E^0_{s1}}}^*)\mathbf{E^0_{spp}} \exp[-i(2k_{spp}r_{xy} - \mathbf{k_{s1}}\cdot\mathbf{r_{xy}})] + (\mathbf{E^{0*}_{spp}}\cdot\mathbf{E^0_{s1}})\mathbf{E^0_{spp}} \exp(-i\mathbf{k_{s1}}\cdot\mathbf{r_{xy}})+$$
$$+(\mathbf{E^0_{spp}}\cdot{\mathbf{E^0_{s2}}}^*)\mathbf{E^0_{spp}} \exp[-i(2k_{spp}r_{xy} - \mathbf{k_{s2}}\cdot\mathbf{r_{xy}})] + (\mathbf{E^{0*}_{spp}}\cdot\mathbf{E^0_{s2}})\mathbf{E^0_{spp}} \exp(-i\mathbf{k_{s2}}\cdot\mathbf{r_{xy}})+ \tag{S5}$$
$$+(\mathbf{E^0_{s1}}\cdot{\mathbf{E^0_{s2}}}^*)\mathbf{E^0_{spp}} \exp[-i(2k_{spp}r_{xy} + \mathbf{k_{s1}}\cdot\mathbf{r_{xy}} - \mathbf{k_{s2}}\cdot\mathbf{r_{xy}})]+$$
$$+({\mathbf{E^0_{s1}}}^*\cdot\mathbf{E^0_{s2}})\mathbf{E^0_{spp}} \exp[-i(2k_{spp}r_{xy} + \mathbf{k_{s2}}\cdot\mathbf{r_{xy}} - \mathbf{k_{s1}}\cdot\mathbf{r_{xy}})]$$

The radiative part of the reconstructed field distribution, $\mathbf{E_{sc}}(\mathbf{x},\mathbf{y})$, contains two terms (terms with $\mathbf{k_{s1}},\mathbf{k_{s2}}$) representing the signal waves [Figure S2(b)]. Additionally, a part of the radiated light is diffusely scattered, also propagating away from the surface (the last two terms). However, in our case of **orthogonally polarized** signal waves, the dot products of the two signal field amplitudes become zero. Therefore, once again only the signal waves are propagating away in the process of hologram reconstruction:

$$\mathbf{E_{sc}}(\mathbf{x},\mathbf{y}) \sim I(x,y)\mathbf{E_{spp}} \sim (\mathbf{E^{0*}_{spp}}\cdot\mathbf{E^0_{s1}})\mathbf{E^0_{spp}} \exp(-i\mathbf{k_{s1}}\cdot\mathbf{r_{xy}})+$$
$$(\mathbf{E^{0*}_{spp}}\cdot\mathbf{E^0_{s2}})\mathbf{E^0_{spp}} \exp(-i\mathbf{k_{s2}}\cdot\mathbf{r_{xy}}) \tag{S6}$$

S1.3 Cylindrically diverging SPP as a reference wave

Now, we elaborate further on the form of the reference SPP wave, which is a QE-excited radially divergent SPP wave:

$$\mathbf{E_{spp}} = \frac{1}{\sqrt{r_{xy}}}\mathbf{A^0_{spp}}\exp(-\alpha r_{xy})\exp(-i\mathbf{k_{spp}}\cdot\mathbf{r_{xy}}) \tag{S7}$$

where $\alpha = \frac{1}{2L_{spp}}$, and L_{spp} is the SPP propagation length. The SPP wavenumber $k_{spp} = \frac{2\pi}{\lambda}N_{spp}$, where N_{spp} is SPP effective mode index.

One can observe that the SPP attenuates during its propagation due to its radial divergence ($\frac{1}{\sqrt{r_{xy}}}$) and absorption ($\exp(-\alpha r_{xy})$). In the process of the hologram reconstruction, the SPP is also depleted owing to its out-of-plane scattering by hologram nanoparticles. The above factors limit the domain of interference and even more so the hologram area efficiently used in the reconstruction. Considering the above SPP field (eq. S7) used as the reference wave during the hologram recording and reconstruction, the reconstructed signal wave term (similar to eq. S3) becomes:

$$\mathbf{E_{sc}} \sim I\mathbf{E_{spp}} \sim (\mathbf{A^{0^*}_{spp}}\cdot\mathbf{E^0_s})\mathbf{A^0_{spp}}\frac{1}{r_{xy}}\exp(-2\alpha r_{xy})\exp[-i\mathbf{k_s}\cdot\mathbf{r_{xy}}] \tag{S8}$$

Here, the inverse radially and exponentially decaying factors appear, limiting the hologram spatial domain used for the signal reconstruction to a small area around the QE and making faithful reconstruction rather problematic. This problem can however be circumvented, if we compensate these decaying terms at the stage of hologram recording, when the latter is implemented by calculating the interference pattern and fabricating the corresponding (computer-generated) hologram, by using the **artificially growing** SPP field, i.e., calculating the interfence pattern $I_a(x,y) = \left|\mathbf{E_{a,spp}} + \mathbf{E_s}\right|^2$, where:

$$\mathbf{E_{a,spp}} = \sqrt{r_{xy}}\mathbf{B^0_{spp}}\exp(\alpha r_{xy})\exp(-i\mathbf{k_{spp}}\cdot\mathbf{r_{xy}}) \tag{S9}$$

This compensation contrivance allows one to reconstruct the signal wave, which is relatively

faithful as far as the amplitude and phase distribution near the array of hologram nanoparticles is concerned (although leaving the problem of polarization reconstruction unresolved):

$$\mathbf{E_{sc}} \sim I_a \mathbf{E_{spp}} \sim (\mathbf{B^{0^*}_{spp}} \cdot \mathbf{E^0_s})\mathbf{A^0_{spp}} \exp[-i\mathbf{k_{spp}} \cdot \mathbf{r_{xy}}] \tag{S10}$$

where the SPP decay factors are compensated, and the spatial domain of the reconstruction is not explicitly limited. The SPP depletion because of the scattering by the hologram nanoparticles can be compensated by inserting the additional decay term α_{scat} in the attenuation coefficient $\alpha^* = \alpha + \alpha_{scat}$. It should however be borne in mind that the reconstruction domain is implicitly limited by the requirement of hologram nanoparticles being of subwavelength sizes: the exponential growth of the SPP field used in the hologram recording requires a similar growth of the hologram nanoparticle volumes.

Our holographic approach developed for designing the QE-coupled metasurfaces allows one to generate the QE emission with arbitrary wavefronts. We have used this approach to calculate the metasurface pattern for generating two angular-resolved emission channels with two orthogonal linear polarizations. First, the xy-plane field components are calculated for the desired signal beams. Second, the interference pattern is calculated as described above. Finally, the obtained interference distribution $I(x, y)$ is discretized into an array of scatterers, which constitute the dielectric metasurface.

S2 Calculation of metasurface pattern for reconstruction of two signal waves of orthogonal linear polarizations

As described in the previous section, to calculate the metasurface pattern, we first calculate the interference pattern of the **two signal waves** and the **artificial SPP** wave and further discretize it into a binary pattern with pixels either filled with dielectric or kept empty. The wavelength of the light is set to $\lambda_0 = 602$ nm to match the GeV emission peak.

The calculation is two-dimensional, and all the fields are projected onto the xy-plane. The field components, which are normal to the hologram (metasurface) plane, are disregarded, because their scattering along directions close to the normal is negligibly small (within our electric-dipole approximation). The obtained metasurface pattern is also two dimensional being associated with the two-dimensional intensity interference of signal and reference waves.

The considered system is a planar interface between silver coated with 30 nm SiO_2 and air oriented in the xy-plane.

Two Gaussian beams of orthogonal linear polarizations are considered as signal waves:

$$\mathbf{E_s^x}(x,y) = \mathbf{E^0}\exp(-\frac{r^2}{\omega_0^2})\exp[-i\mathbf{k_s^x}\cdot\mathbf{r}] \tag{S11}$$

$$\mathbf{E_s^y}(x,y) = \mathbf{E^0}\exp(-\frac{r^2}{\omega_0^2})\exp[-i\mathbf{k_s^y}\cdot\mathbf{r}] \tag{S12}$$

The first signal beam has the polarization along the x-axis and the propagation direction $\mathbf{k_s^x}$ in the zy-plane, beam waist ω_0. The deflection angle from the z-axis is $\theta = 15°$ as illustrated in Figure S3. The second signal beam has the polarization along the y-axis and the propagation direction $\mathbf{k_s^y}$ in the zx-plane with a 15° deflection from the z-axis. The projections of the signal waves in the xy-plane are as follows:

$$E_s^x(x,y) = E^0 cos(\theta)\exp(-\frac{r^2}{\omega_0^2})\exp[-ik_0 x sin(\theta)] \tag{S13}$$

$$E_s^y(x,y) = E^0 cos(\theta)\exp(-\frac{r^2}{\omega_0^2})\exp[-ik_0 y sin(\theta)] \tag{S14}$$

As discussed in the section S1.3, artificially increasing the SPP wave(eq. S9) is used to compensate for the SPP divergence and absorption (and eventually scattering) and, in doing so, to eliminate the spatial limitation of the domain of its interference with the signal waves. The artificially growing SPP wave is radially divergent from the origin, $(x,y) = (0,0)$:

$$\mathbf{E}_{a,spp} = \sqrt{r_{xy}}\mathbf{B_{spp}^0}\exp(\alpha r_{xy})\exp(-i\mathbf{k_{spp}}\cdot\mathbf{r_{xy}}) \tag{S15}$$

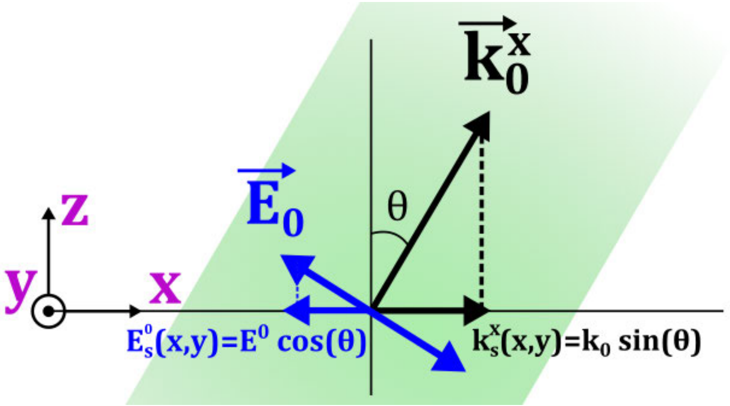

Figure S3: Projection of the X-polarized signal wave onto the XY plane. For interference pattern calculation the signal wave (field $\mathbf{E_0}$ polarized along X-axis (blue), wavevector $\mathbf{k_0^x}$) is projected onto XY plane. The Y-polarized signal wave is projected in the similar manner. Angle θ defines the inclination of the signal wave in respect to the normal to the hologram plane and direction of the reconstructed wave.

where, $k_{spp} = k_0 N_{eff}$ is SPP propagation constant, $k_0 = 2\pi/\lambda_0$ the wave vector of free-space propagating wave, $\lambda_0 = 602$ nm. $\mathbf{r_{xy}}$ denotes the radius vector. The SPP effective mode index N_{eff} is calculated as follows:

$$N_{eff} = (1-\chi)N_{eff}(air) + \chi N_{eff}(HSQ) \tag{S16}$$

The effective mode indices for the Ag/SiO_2/HSQ and Ag/SiO_2/air interfaces are numerically calculated separately as $N_{eff}(air) = 1.12$ and $N_{eff}(HSQ) = 1.52$, respectively, resulting in $N_{eff} = 1.32$. The filling factor χ was fixed at 50%, denoting the dielectric/air ratio of the metasurface. The fixed filling factor allowed simplification of the calculations by utilizing the a constant SPP mode index for the structure. The refractive indices of the dielectrics were set to $n(SiO_2)$ = 1.45 and $n(HSQ) = 1.41$. The SPP decay factor $\alpha_{abs} = \frac{1}{2L_{spp}} = Im\{k_{spp}\}$. In our experiment, the SPP propagation length is L_{spp} =17 µm and $\alpha_{abs} = 0.03$. The Johnson&Christy[S2] silver optical constants were used for pattern generation. Also, calculations based on Palik[S3] optical constants of silver were performed for a comparison (Figure S6).

Next, the interference between the reference $\mathbf{E}_{a,spp}$ and the two signal waves $\mathbf{E_s^x}, \mathbf{E_s^y}$ is calculated as described above. Note that intensity interference patterns are typically composed

of interference fringes. According to our scattering holography approach for recording the hologram, one should therefore arrange (spherical) nanoparticles along the lines (interference fringes) varying their volumes in accordance with the local intensity levels. Such an approach although being feasible (and in line with the theoretical foundation described above) is extremely demanding as far as practical implementation is concerned. Targeting the conventional fabrication, for example with the electron-beam lithography, we consider the nanoparticles being fused into continuous nanoridges of the constant height but with the local width being proportional to the local intensity in the interference pattern. This approach is adopted throughout the present work, resulting in high quality and efficient reconstruction of signal waves by QE-excited SPP being scattered out with the designed metasurfaces, as demonstrated in this work.

S2.1 Initial design

For the first approximation, the hologram recording and reconstruction processes can be built under the following assumptions:

1. Constant SPP amplitude, neglecting the SPP field attenuation due to divergence factor $[\frac{1}{\sqrt{r_{xy}}}]$, absorption $[\exp(-\alpha r_{xy})]$ and depletion owing to its scattering by hologram nanoparticles.

$$\mathbf{E_{a0,spp}} = \mathbf{A^0_{spp}} \exp(-i\mathbf{k_{spp}} \cdot \mathbf{r_{xy}}) \tag{S17}$$

2. Plane, linearly polarized signal waves.

$$E^x_{s,plane}(x, y) = E^0 cos(\theta) \exp[-ik_0 x sin(\theta)] \tag{S18}$$

$$E^y_{s,plane}(x, y) = E^0 cos(\theta) \exp[-ik_0 y sin(\theta)] \tag{S19}$$

The interference patterns $I(x, y)$ that emerge from the interference between $\mathbf{E_{a0,spp}}$ and projections of signal wave fields $E^x_{s,plane}$ and $E^y_{s,plane}$ are shown in Figures S4(a-b). The final interference pattern of all three waves is shown in Figure S4(c).

Next, we discretize the obtained hologram pattern to generate the design pattern suitable for the fabrication with the electron-beam lithography (as discussed in the preceding section). The field intensity distribution is normalized and lies within the rang e [0–1]. The threshold value is defined by the structure filling factor (in our case $\chi = 0.5$). In this case, half of the metasurface area is filled with material. Therefore, half of the area of the interference pattern should be below the threshold and half should be above. Thus we find the value of threshold and discretize the structure treating all pixels larger than threshold as "1" and, oppositely, all pixels smaller than threshold as "0." The binary image is shown in Figure S4(h). All "1" pixels correspond to the positions of noninteracting isotropic scatterers. In our case, these points were filled with the HSQ. The calculated far-field distributions presented in Figure S4(e-g) show the generation of two separate beams with orthogonal polarizations.

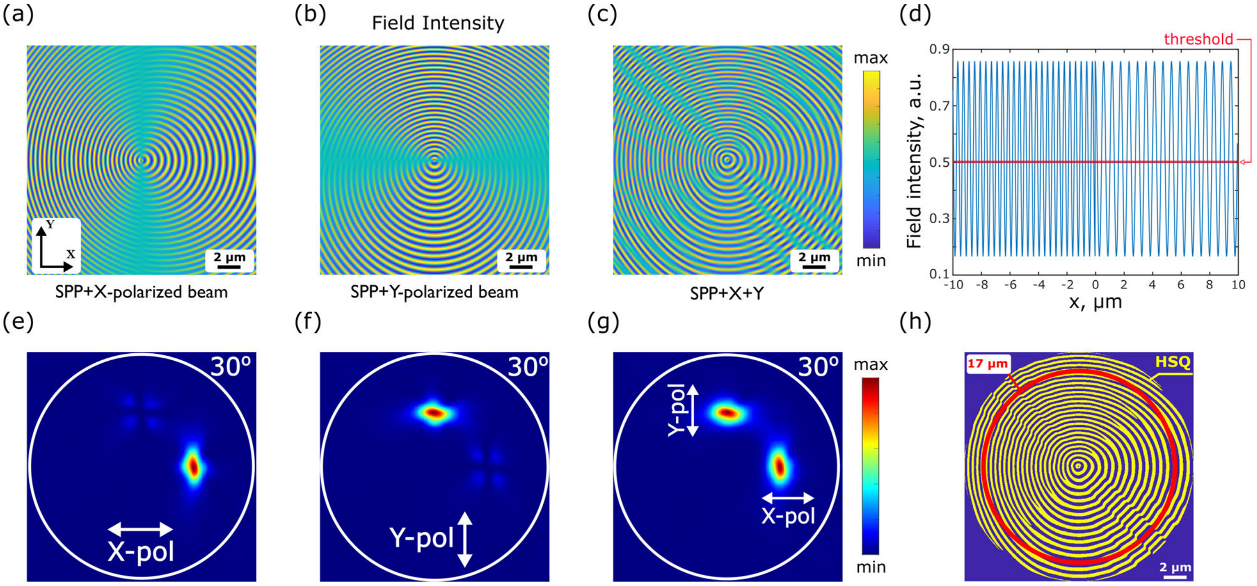

Figure S4: Initial design of the hologram metasurface. Interference between SPP and X- or Y- polarized beam fields on the sample surface is shown in (a) and (b), respectively. The final interference pattern (c) between SPP, X and Y polarized beams. (d) The cross section of (c) with the threshold used for discretization. (h) Binary image of metasurface suitable for the electron beam lithography. (e-g) Simulated far-field patterns representing emission distributions inside the 30° cone.

Therefore, these initial approximations are sufficient to design a metasurface that produces two separate beams of orthogonal linear polarziations, as presented in Figure S4(e-g). The external quantum efficiency $QE = 63\%$ for the metasurface with $D = 17$ μm diameter shown in the Figure S6, column "Initial design." However, the collimation and polarization clarity of the produced beams [S6(b)] can be improved, as we show in the next section.

S2.2 Optimized design

To elevate the beam quality in terms of collimation and polarization clarity, the Gaussian profile of the signal beams and depletion of the reference SPP wave are considered.

The Gaussian profile of the signal waves is represented by the $\exp(-r^2/\omega_0^2)$ term in field $E_{x(y)}$ of the beam (eq. S14). The beam waist, ω_0, becomes an important optimization parameter because it limits the spatial domain of the interference pattern calculation, as shown by Kan et al.[S1].

$$E_s^x(x,y) = E^0 \exp(-\frac{r^2}{\omega_0^2}) cos(\theta) \exp[-ik_0 x sin(\theta)]$$
$$E_s^y(x,y) = E^0 \exp(-\frac{r^2}{\omega_0^2}) cos(\theta) \exp[-ik_0 y sin(\theta)]$$

As discussed above, to compensate and enlarge the interference calculation domain, we utilize an artificial SPP field that grows with distance from the QE [S15]:

$$\mathbf{E}_{a,spp} = \sqrt{r_{xy}} \mathbf{B}^{\mathbf{0}}_{\mathbf{spp}} \exp(\alpha r_{xy}) \exp(-i \mathbf{k}_{\mathbf{spp}} \cdot \mathbf{r}_{\mathbf{xy}})$$

To obtain the optimized hologram pattern we repeat the interference pattern calculation and discretization described in the previous section with new signal $\mathbf{E}^{\mathbf{x}}_{\mathbf{s}}, \mathbf{E}^{\mathbf{y}}_{\mathbf{s}}$ and reference $\mathbf{E}_{a,spp}$ waves.

The performance of the metasurface depends on the contrast of the interference pattern. The largest contrast can be obtained when the amplitudes of the signal and reference fields are equal to each other. Therefore, we fixed the SPP amplitude and varied the signal waves amplitudes and beam waist to find the pattern with the maximum contrast [Figure S5(a)]. The discretization is performed as described in the previous section, with the threshold as a point where half of the area of the interference pattern is above that point [Figure S5(b)].

The resulting discretized metasurface pattern has widened outer ridges that increase their scattering power, thus compensating the SPP radial decay [Figure S5(c)]. The SPPs are scat-

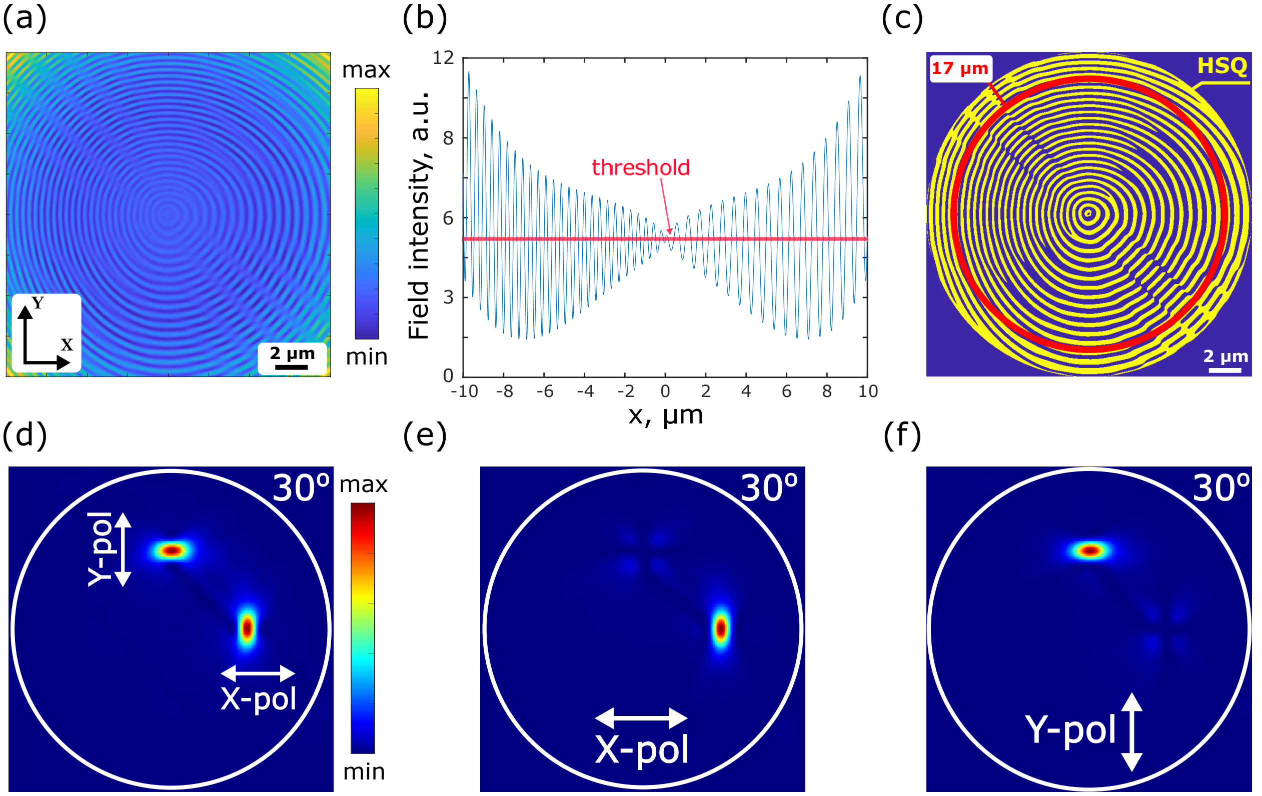

Figure S5: Optimized design of the hologram metasurface. (a) The interference pattern between artificial SPP, x- and y- polarized Gaussian beams. (b) The cross section of (a) with the threshold used for discretization. (c) Binary image of metasurface suitable for the electron beam lithography. (d-f) Simulated far-field patterns representing emission distributions inside the 30° cone.

tered by the metasurface structure and absorbed by the metal. Wider outer ridges increase the scattering, thereby shortening the effective SPP propagation distance and decreasing the SPP absorption, i.e., increasing the efficiency of the metasurface operation. However, with further increase in the radius, ridges start to merge (manifesting the aforementioned implicit limitation), and we cut the metasurface extension at that point, because the SPP scattering disappears once the ridges are merged in a continuous film.

This procedure results in improved beams collimation, as shown in Figures S6(d-f).

S2.3 Performance comparison of initial and optimized metasurface design

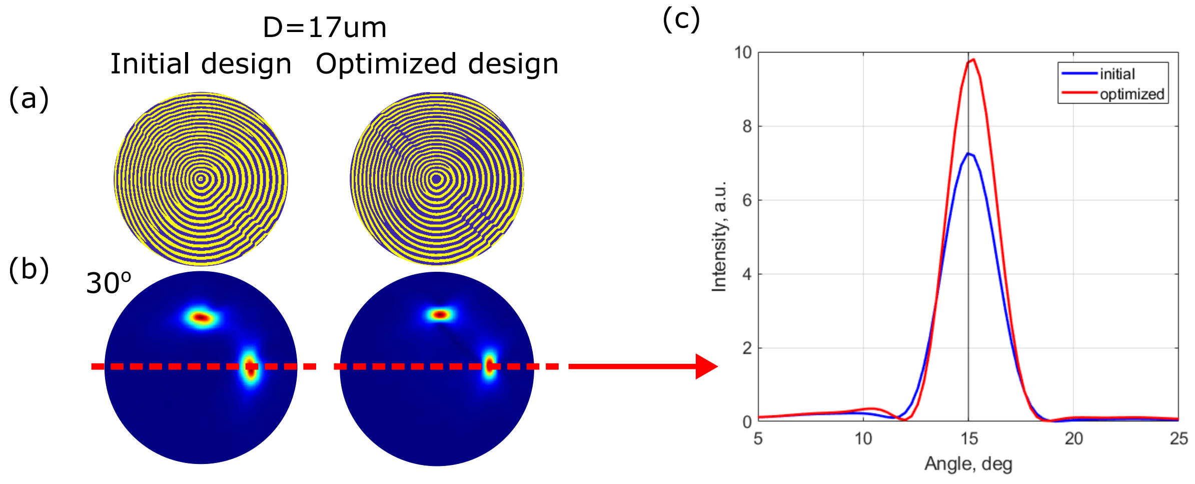

(d)

63%	63%	QE (J&C)
45%	45%	QE (Palik)
17.4%	12.8%	Overlap integral
19	10	Peak area (a.u.)
2.9	2.3	Beam FWHM $\theta^{X}_{1/2}$
7.2	5.2	Beam FWHM $\theta^{Y}_{1/2}$

(e)

NA	CE(init)	CE(opt)
0.3	57%	67%
0.5	74%	85%
0.9	94%	98%

Figure S6: Comparison of the performance of the initial and optimized metasurfaces (a) Design of the metasurfaces. (b) Improvement of the beam collimation in resulting far-field emission patterns. Comparison of the beam profiles clearly shows higher peak intensity and better collimation of the optimized beam (c). Table (d) shows results of optimization in terms of primary metasurface parameters. The comparison of the Collection Efficiency (CE) for 0.3, 0.5 and 0.9 NA objectives is displayed in table (e).

A comparison of the emission characteristics of the initial and optimized metasurface design is presented in Figure S6. The optimized structure is designed with consideration to Gaussian profiles of the signal beams and compensation for SPP decay. As a result, the reconstructed signal beams are significantly better collimated, featuring lower overlap and stronger peak intensities [Figure S6(b,c)].

The first parameter of comparison is the quantum efficiency calculated (for details of calcula-

tion - see section S3) using the Johnson & Christy[S2] and Palik[S3] optical constants for the silver layer of the substrate. The optimized structure exhibits 63% and 45% quantum efficiencies for Johnson & Christy and Palik data, respectively. The quantum efficiency value is lower for the Palik case because of the higher absorption in silver. As expected, the external quantum efficiency of the optimized device is not enhanced because there is no change in the energy contained in the SPP wave, and the energy is only redirected in a more collimated way. The next parameter is the overlap integral, which is a figure-of-merit for beams' separation. The lower the value of the overlap integral, the better the splitting of the beams. The overlap integral is calculated as

$$\text{Overlap} = \frac{[\iint \sqrt{I_x} \cdot \sqrt{I_y} dk_x dk_y]^2}{\iint I_x dk_x dk_y \cdot \iint I_y dk_x dk_y} \tag{S20}$$

where I_x and I_y are the intensities of the x- and y-polarized emission, respectively, and integration is performed over a 30° emission cone, which is projected to the far-field as a disc. The integration area contains both emission lobes. The peak area measurement allows us to conveniently compare the beam collimation efficiency. The area of the peaks is calculated by counting all the pixels of the far-field emission distribution with an intensity higher than the 10% threshold. The divergence angle $\theta_{1/2}^{X(Y)}$ is calculated as angular y-polarized beam full width at half maximum along the x(y) direction. We consider only the y-polarized beam because beams are symmetrical relative to the structure diagonal.

From the comparison, we observe that the beams divergence is improved from 4.3° to 3.2° (S6(d)). The overlap integral is reduced from 17.4% to 12.8%. This leads to a significant enhancement in collection efficiency for low-NA objectives (0.3, 0.5 NA) (S6(e)). The CE is calculated as the ratio of power emitted in a certain NA to the power emitted in 90° (NA = 1).

S2.4 Performance comparison of metasurfaces of different sizes

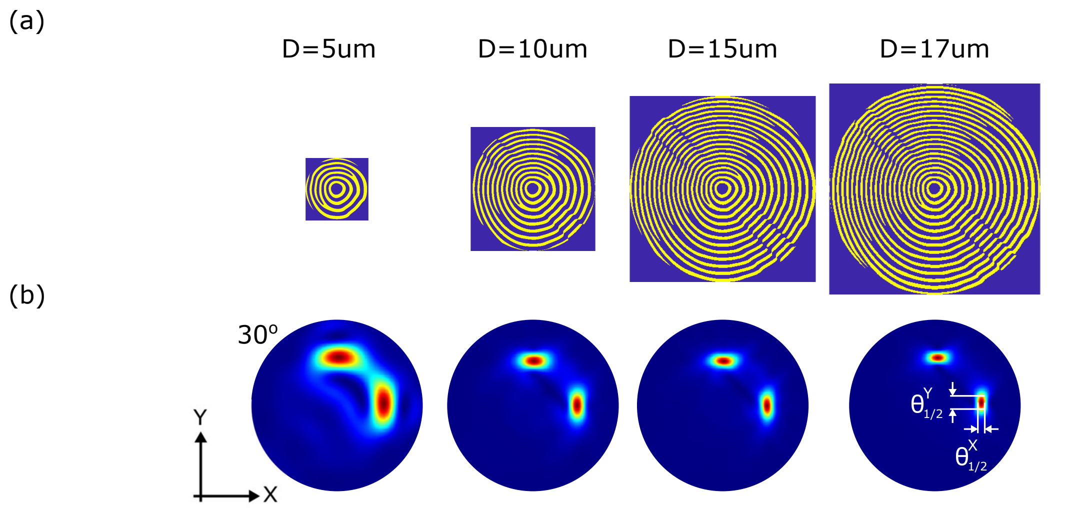

(c)

	D=5um	D=10um	D=15um	D=17um
Quantum efficiency (J&C)	43%	54%	61%	63%
Quantum efficiency (Palik)	32%	41%	44%	45%
Overlap integral	17.4%	13.7%	12.9%	12.8%
Peak area (a.u.)	49	15	11	10
Beam FWHM $\theta^X_{1/2}$	6.3	3.7	2.6	2.3
Beam FWHM $\theta^Y_{1/2}$	13.3	7.5	5.5	5.2

Figure S7: Metasurface size comparison. (a) Top view of metasurfaces with corresponding diameters of 5, 10, 15, and 17 µm. (b) Calculated far-field patterns representing emission distribution inside 30° cone. The increase of the structure diameter enhances the beam collimation. (c) Comparison table of metasurface performance.

The dependences of the calculated holographic metasurface performances on its size are presented in Figure S7. All holographic patterns with diameters 5, 10, 15, and 17 µm were cut from the same D=20 µm structure with the optimized design.

S3 Quantum efficiency calculation

For a quantum emitter coupled to a metasurface, the quantum efficiency can be written as:

$$\eta_{QE} = \eta_{IQE}\eta_{EQE} \tag{S21}$$

where η_{QE} denotes quantum efficiency of the coupled system, η_{IQE} denotes the internal quantum efficiency of the quantum emitter, and η_{EQE} denotes the external quantum efficiency of the coupled system. η_{IQE} depends on the properties of the emitter and can vary between 0 and 1 for different emitters. η_{EQE} depends on the device, in our case, a metasurface. With η_{IQE} assumed unity for an emitter, the quantum efficiency of the coupled system will be equal to external quantum efficiency. In this section, we present a method that we have utilized to calculate the external quantum efficiency of our device.

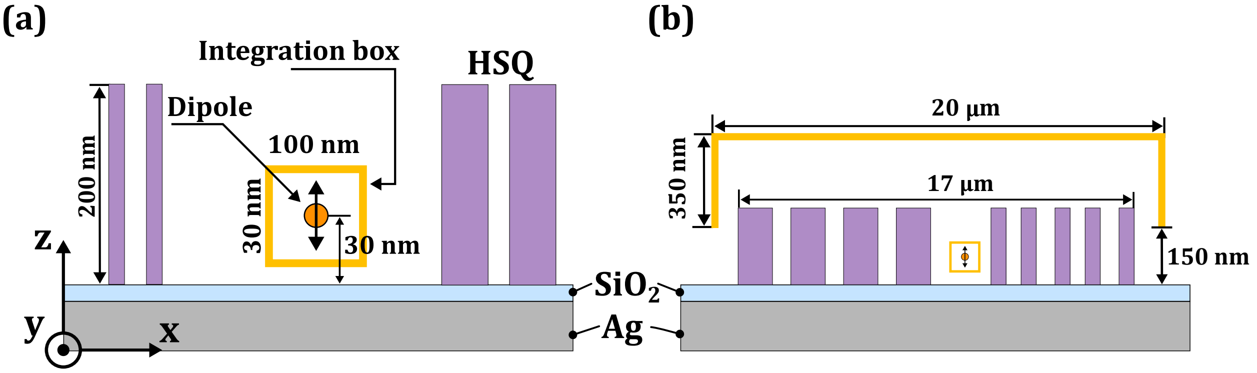

Figure S8: Calculation of external quantum efficiency. The power radiated by the point dipole was calculated by integrating the Poynting vector over the 100x100x30 nm (x,y,z) box around the dipole (a). The power of the outcoupled emission was calculated by integrating the Poynting vector over a 20x20x0.35 µm cap on top of the metasurface (b). The ratio of the two powers is the external quantum efficiency. The thickness of the SiO_2 layer is 30 nm. The thickness of the Ag layer is 320 nm.

The external quantum efficiency was calculated numerically using an FDTD software. η_{QE} was calulated as the ratio between the power radiated into outgoing waves, propagating within a 64° cone (collected by an NA = 0.9 objective) and total power emitted by QE in the presence of the plasmonic environment (metasurface).

The power generated by the dipole in the presence of the plasmonic environment was

calculated by integrating the Poynting vector over a 100x100x30 nm (x,y,z) box enclosing the point dipole. The power outcoupled by the metasurface to the propagating waves was calculated by integrating the Poynting vector over the cuboid cap, covering the metasurface, and placed at a reasonable distance (300 nm) from the top of the metasurface to avoid the influence of the near-field. The size of the cap was 20×20×0.35 μm, covering the metasurface with a diameter of 17 μm. To be consistent with the experimental measurements, the size of the cap was adjusted to count only the radiation emitted in the NA=0.9 (64 deg). This procedure resulted in $\eta_{QE} = 0.63$ external quantum efficiency. Predictions obtained using a similar method were experimentally verified before[S4,S5]. It should be noted that the efficiency can be further increased by using high-refractive index metasurface ridges to boost their out-of-plane scattering and high-quality monocrystalline metal films to reduce the ohmic losses[S6]. Thus, simply by changing the ridge material to titanium dioxide (TiO2), the external quantum efficiency is expected to exceed 80%, even without further optimization of ridge parameters as described for the bullseye pattern[S5].

The quantum efficiency of the coupled system depends on the scattering and propagation loss of SPPs. Below, we present a method to separately estimate the effects of scattering and propagation loss on quantum efficiency.

The SPP energy is either scattered by metasurface ridges or absorbed by metal because of the ohmic losses:

$$P_0 = P_{scat} + P_{abs} \tag{S22}$$

where P_0 is part of the dipole emission power that is coupled to the SPP mode.

So, the outcoupling efficiency can be defined as:

$$\varepsilon_{scat} = \frac{P_{scat}}{P_0} \tag{S23}$$

To estimate P_{scat}, we need to consider the process of the SPP scattering by the metasurface ridges depending on the distance from the SPP source. We consider 2D metal-dielectric interface

(xy-plane). The field of the radially divergent SPP propagating on the clean metal-dielectric interface (without metasurface) is described by:

$$\mathbf{E_{SPP}} = \mathbf{E^0_{spp}} \frac{1}{\sqrt{r}} exp(-\alpha_{abs} r) exp(-i \mathbf{k_{spp}} \cdot \mathbf{r}) \tag{S24}$$

where $\alpha_{abs} = \frac{1}{2L_{spp}}$, with L_{spp} being the SPP propagation length (over which the SPP intensity decreases by $1/e$), and $\mathbf{k_{spp}}$ is SPP wavevector with corresponding wavenumber $k_{spp} = \frac{2\pi}{\lambda} N_{spp}$, with N_{spp} being the SPP effective index at the operating wavelength λ. The radial divergence is represented by the term $\frac{1}{\sqrt{r}}$, where r is the radius. Here α_{abs} is defined for the ohmic losses in metal.

Therefore, the SPP intensity is:

$$I_{spp} = I^0_{spp} \frac{1}{r} exp(-2\alpha_{abs} r) \tag{S25}$$

In the presence of the metasurface the SPP field is also depleted due to scattering by the dielectric ridges. Therefore, the attenuation coefficient of the SPP interacting with metasurface ridges should be changed to:

$$\alpha_{tot} = \alpha_{scat} + \alpha_{abs} \tag{S26}$$

and the SPP intensity will decay faster:

$$I_{spp} = I^0_{spp} \frac{1}{r} exp(-2\alpha_{tot} r) \tag{S27}$$

Assuming that power scattered in the thin ring with the thickness dr is proportional to the SPP intensity:

$$dP_{scat} \sim \alpha_{scat}\, I_{spp}\, 2\pi r\, dr \tag{S28}$$

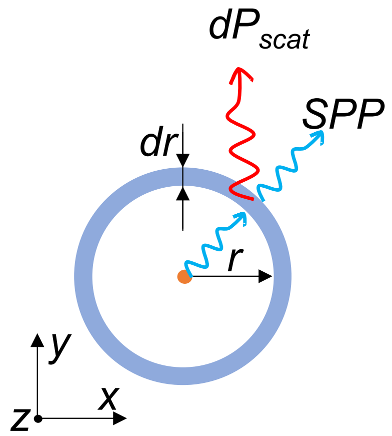

Figure S9: Calculation of the scattered SPP power by dividing the metasurface domain into rings with thickness dr. Each ring located at a distance r from the SPP source (point dipole) scatters power $dP_{scat} \sim \alpha_{scat}\, I_{spp}\, 2\pi r\, dr$. Integration of the dP_{scat}over the metasurface domain $r = [0\ L]$ results in total scattered power by the metasurface P_{scat}.

The total scattered power can be calculated by integrating dP_{scat} over the metasurface area:

$$P_{scat} = \int_0^L \alpha_{scat} I_{spp} 2\pi r\, dr = 2\pi\alpha_{scat} \int_0^L r I_{spp}^0 \frac{1}{r} \exp\left(-2\alpha_{tot} r\right) dr = 2\pi\alpha_{scat} \int_0^L I_{spp}^0 \exp\left(-2\alpha_{tot} r\right) dr \tag{S29}$$

The result is:

$$P_{scat} = \pi I_{spp}^0 \frac{\alpha_{scat}}{\alpha_{tot}} \left(1 - exp\left(-2\alpha_{tot} L\right)\right) \tag{S30}$$

The dependance $P_{scat}(L)$ can be obtained by estimation of the outcoupled power for metasurfaces with increasing diameters in numerical simulations. Using this function to fit the simulated data allows us to estimate the decay coefficient $\alpha_{tot} = 0.13$.

Following a similar procedure, the part of the energy absorbed in metal can be written as:

$$P_{abs} = \pi I_{spp}^0 \frac{\alpha_{abs}}{\alpha_{tot}} \left(1 - exp\left(-2\alpha_{tot} L\right)\right) \tag{S31}$$

The SPP decay coefficient $\alpha_{abs} = 0.03$ was calculated analytically:

$$\alpha_{abs} = \frac{1}{2L_{spp}} = Im\left(k_{spp}\right) = Im\left(\frac{2\pi}{\lambda} N_{spp}\right)$$

In our case $\lambda = 602\ nm$ and $N_{spp} = 1.28 + i \cdot 2.77 \cdot 10^{-3}$ calculated for Ag-SiO_2-HSQ-air interface

with HSQ filling factor 0.5, which corresponds to the metasurface presented in the manuscript.

The SPP decay coefficient attributed to scattering is calculated as:

$$\alpha_{scat} = \alpha_{tot} - \alpha_{abs} = 0.1 \tag{S32}$$

This results in 78% outcoupling efficiency ($\alpha_{scat} = 0.1,\ \alpha_{tot} = 0.13$):

$$\varepsilon_{scat} = \frac{P_{scat}}{P_0} = \frac{P_{scat}}{P_{scat} + P_{abs}} = \frac{\alpha_{scat}}{\alpha_{scat}+\alpha_{abs}} = \frac{\alpha_{scat}}{\alpha_{tot}} = 0.78 \tag{S33}$$

The external quantum efficiency is a product of efficiency of the dipole coupling to SPP η_{spp}, scattering of SPPS by the metasurface ridges η_{scat} and collection efficiency of the objective η_{CE}, that is, $\eta_{QE} = \eta_{spp}\eta_{scat}\eta_{CE}$.

First, the excitation efficiency of SPP waves is $\eta_{spp} > 0.8$ for proper orientation of the dipole placed at a reasonable distance from the metal surface[S7]. This value can also be derived using an analytical model as well[S8]. Second, the scatterd part of the propagating SPP, by the dielectric ridges of the metasurface is calculated to be $\eta_{scat} \approx 0.78$, the remaining 22% are absorbed by the metal. The collection efficiency, defined as the fraction of the total outcoupled power collected by the objective of NA=0.9, was numerically calculated to be $\eta_{CE} = 0.98$. Taking all the aforementioned factors into account, the calculated total external quantum efficiency of is: $\eta_{QE} = 0.8 * 0.78 * 0.98 = 0.61$, which is close to the value simulated numerically.

Using high-refractive index metasurface ridges, for example made of titanium dioxide, would further increase the contribution of the scattering SPP loss, making the SPP absorption loss contribution negligible and reaching the total external quantum efficiency of 0.8 (as was obtained for the bullseye pattern[S5].

S4 Metasurface function robustness against shift of the ND

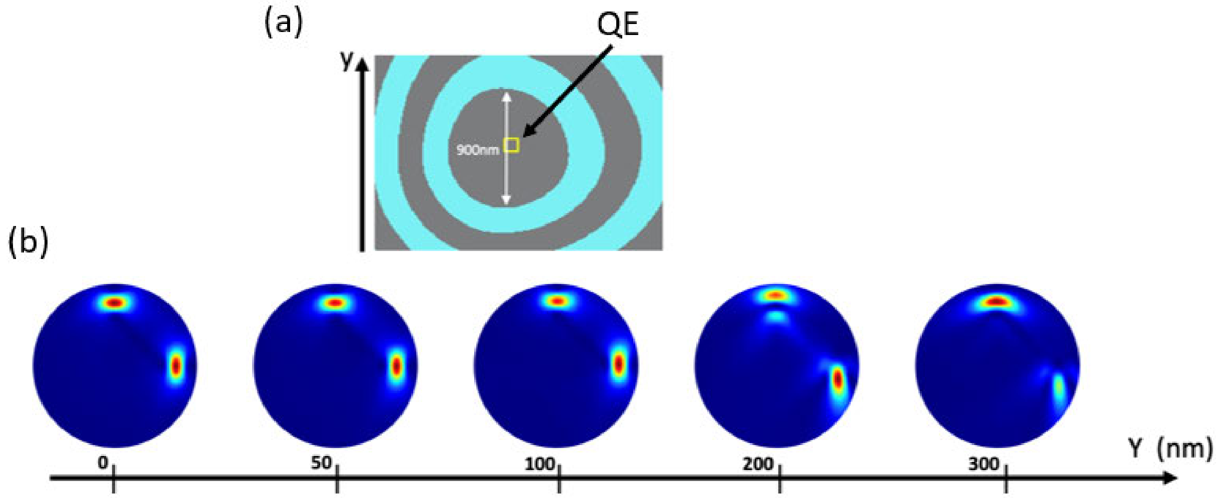

Figure S10: Metasurface function robustness against ND shift from the center.(a) Metasurface top view.(b) Far-field patterns of emission generated by source with the QE shifted by up to 300 nm from the central position along y-axis. From the simulation results, we observe that shifts smaller than 100 nm have no significant effect on the emission pattern.

To estimate the sensitivity of the metasurface properties on the ND shift from the central position, we perform numerical simulations analyzing changes in the far-field pattern of the metasurface emission caused by ND displacement. As we can see in Figure S10, 50 and 100 nm shifts along the y-axis do not change the far-field intensity distribution and cause only an imbalance in beam amplitudes. 200 nm shift of the ND results in visible distortion in the shape of both beams. It also causes a shift in positions and splitting of one of the beams into two. However, the metasurface still performs its function of generation of two channels of orthogonal linear polarizations. 300 nm and larger shifts lead to a significant deviation from the unperturbed state with largely reduced amplitude of one of the beams. Overall, we expect stable metasurface operation within 100 nm ND shifts which corresponds to the unavoidable error in our positioning technique.

S5 Metasurface fabrication

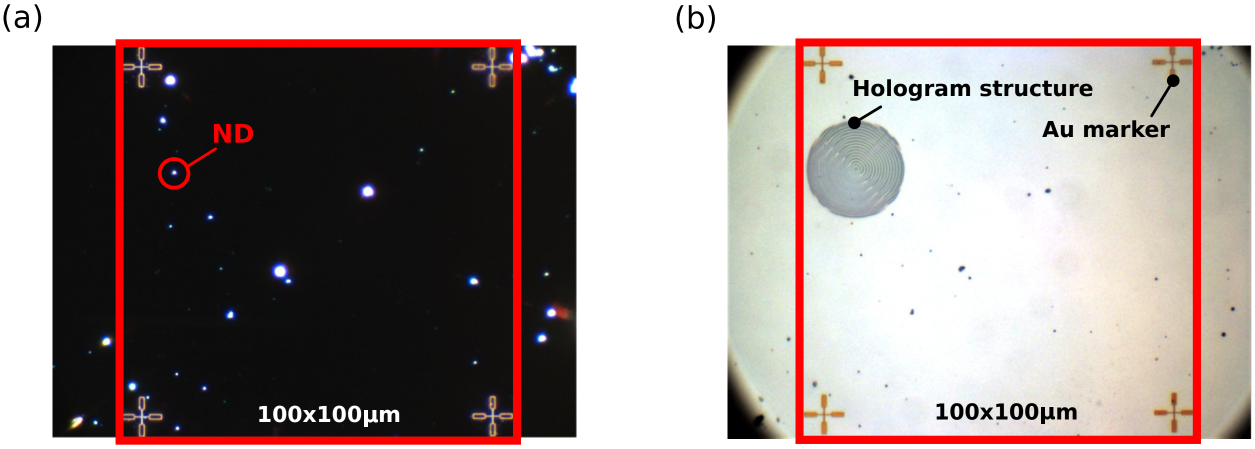

Figure S11: Positioning of the holographic metasurface. (a) Dark field image of deposited nanodiamonds. (b) Bright field image of the palsmonic hologram metasurface fabricated around chosen nanodiamond. 100x magnification.

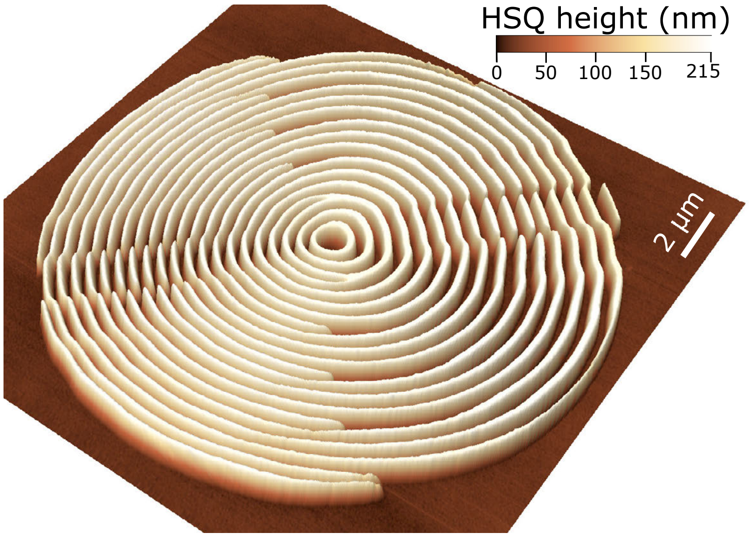

Figure S12: 3D atomic force microscopy scan image of the hologram metasurface. The sample was measured in the non-contact mode with a scan area of 20 µm × 20 µm.

We fabricated our samples in following five steps:

1. Substrate preparation: A polished Si wafer is coated by thermal evaporation of 3 nm Ti followed by 150 nm Ag, and another 3 nm Ti. Radio frequency magnetron sputtering of 30 nm SiO_2 layer is done to protect silver from oxidation. Ti layer is used for better

adhesion between Si and SiO_2 to metal. Deposition rates for Ti/Ag/Ti/SiO_2 structure are 0.1/1/0.1/1.5 Å/s, respectively. Chamber pressure used for depositions is $\sim 5 \cdot 10^{-6}$ mbar.

2. Gold markers fabrication by electron beam lithography: PMMA A2 positive resist is spin-coated at 1580 rpm for 45 sec and baked at 180°C on a hot plate for 2 min to form a ≈100 nm layer. 10x10 μm^2 crosses are patterned using an electron beam lithography (EBL) system and developed for 35 sec in 1:3 MIBK:IPA (Methyl isobutyl ketone:Isopropyl alcohol) and rinsed in IPA for 60 sec. Ti(3nm)/Au(35nm) layers are thermally deposited in a vacuum chamber with deposition rates 0.1/1 Å/s and pressure $5 \cdot 10^{-6}$ mbar. The unexposed PMMA is dissolved in acetone during a 12 hours lift-off procedure. Samples are ultrasonicated in acetone for 1 min and washed with IPA and DI water. Gold is used as a material for markers because it is chemically stable and provides sufficient contrast for optical microscopy and fluorescent mapping.

3. Nanodiamond deposition: To stabilize nanodiamonds on the surface we cover the substrate with an adhesive Poly(allylamine hydrochloride). Further, a water dispersion of Nanodiamonds containing Germanium vacancy centers (GeV-NDs) with average size ~100 nm size is spin-coated on the sample. The positions of diamonds are determined relative to the gold markers using dark-field microscope images shown in Figure S11(a). The positioning accuracy is ≈50 nm. The positioning procedure is described in previous work[S4]. The procedure of GeV-ND fabrication is described elsewhere[S9]).

4. Nanodiamond choice: The crucial part of QE-SPP coupled metasurface fabrication is to ensure that the QE radiative transition dipole is predominantly oriented perpendicular to the sample surface, since this results in a high QE-SPP coupling efficiency along with axially symmetric propagating SPPs[S4,S5,S10]. To identify NDs with properly oriented GeV centers, we used a strongly focused and radially polarized excitation beam, producing a strong E_z component of the electric field in the focal point and consequently efficiently exciting GeV centers having a predominant out of plane component of the dipole moment. In the

last step, we fabricate HSQ holographic metasurfaces around individual GeV-NDs using their coordinates determined relative to the markers to properly conduct electron-beam lithography (EBL).

5. Hydrogen silsesquioxane (HSQ) structure fabrication: Negative resist HSQ (Applied Quantum Materials, Canada, 8% solution in MIBK) is spin-coated at 1200 RPM 60 sec and baked at 160°C on a hot plate for 2 min to form a ≈200 nm layer. The metasurface structures are patterned around GeV-NDs by EBL and developed in 25% TMAH (Tetramethylammonium hydroxide) for 4 min. Samples are rinsed for 1 min with DI water. Images of a fabricated structure are shown in Figure S11(b) and Figure S12.

S6 Experimental setup

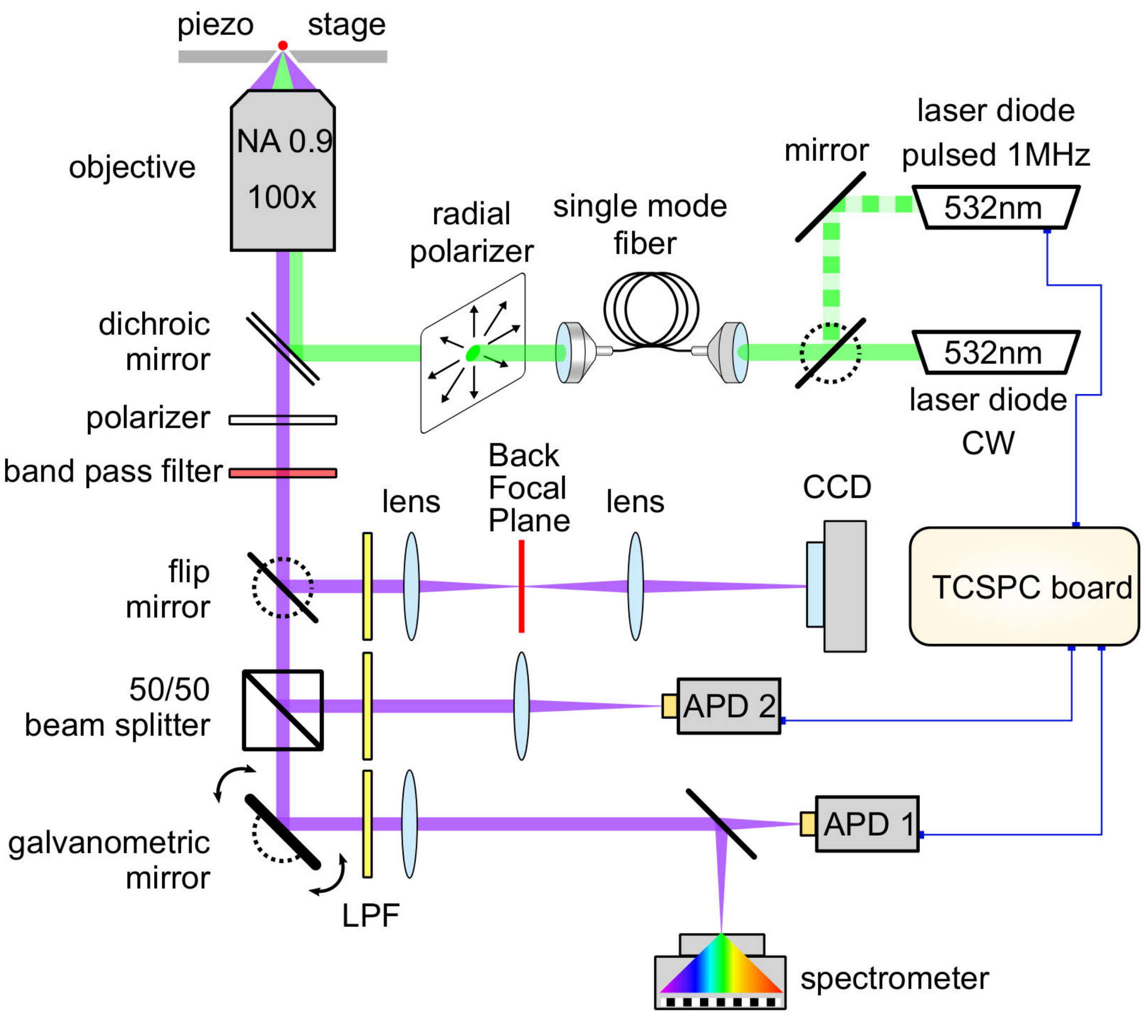

Figure S13: Schematic of experimental setup. CCD — camera. APD — Avalanche Photodiode. TCSPC — Time Correlated Single Photon Counting board. LPF - 550 nm long pass filter.

An experimental set-up schematic is presented in Figure S13. A linearly polarized 532 nm continuous wave (CW) or pulsed laser beam is passed through the single mode polarization maintaining fiber to purify the beam mode. After being outcoupled, the pure linearly polarized beam is converted to radially polarized beam by an ARCoptix RPC Radial Polarization Converter. The Olympus MPLFLN x100 objective with 0.9 NA focuses the radially polarized laser beam on the surface of the sample. Quantum emitters, in our case — GeV-

NDs or SiV-NDs (nanodiamonds containing silicon vacancy centers) are excited by a strong normal to the surface field component in the focal spot. Fluorescence scans are performed by a synchronous movement of the piezo-stage with mounted sample and measuring counts of the collected fluorescence emission on the APD. Laser light is filtered out by a set of Semrock FF535-SDi01/FF552-Di02 dichroic mirrors and pair of Thorlabs FELH0550 550 nm long-pass filters in each detection branch. Fluorescence spectra are measured using an Andor Ultra 888 USB3–BV spectrometer operating within 550–850 nm. Fluorescence decay-rate measurements are carried out using Time Correlated Single Photon Counting (TCSPC) technique with a PicoHarp 300 TCSPC module correlating signals from both Pico Quant LDH-P-FA-530L pulsed excitation laser and PicoQuant τ-SPAD APD. The auto-correlation function is measured with Swabian TimeTagger 20 correlator from two similar PicoQuant τ-SPAD APDs with CW excitation of the ND. The saturation curves of the GeV emission are measured by the accumulation of counts from both APDs with excitation laser power in the range from 10 μW to 7 mW on the sample surface. The back focal plane images are obtained with a Hamamatsu Orca LT+ CCD camera. The Thorlabs GTH10M Glan-Thompson polarizer is inserted in the optical path for measurement of the emission polarization properties. Spectrally filtered images are taken with a 605±8 nm bandpass filter for GeV-NDs and 740±16 nm filter for SiV NDs. The SEM images are obtained with a 30-kV JEOL-6490 electron microscope. The AFM imaging is performed with the NT-MDT NTEGRA atomic force microscope. All experiments are carried out at standard conditions.

S7 Experiment repeatability

S7.1 Characterization of hologram metasurface coupled to another single-photon GeV

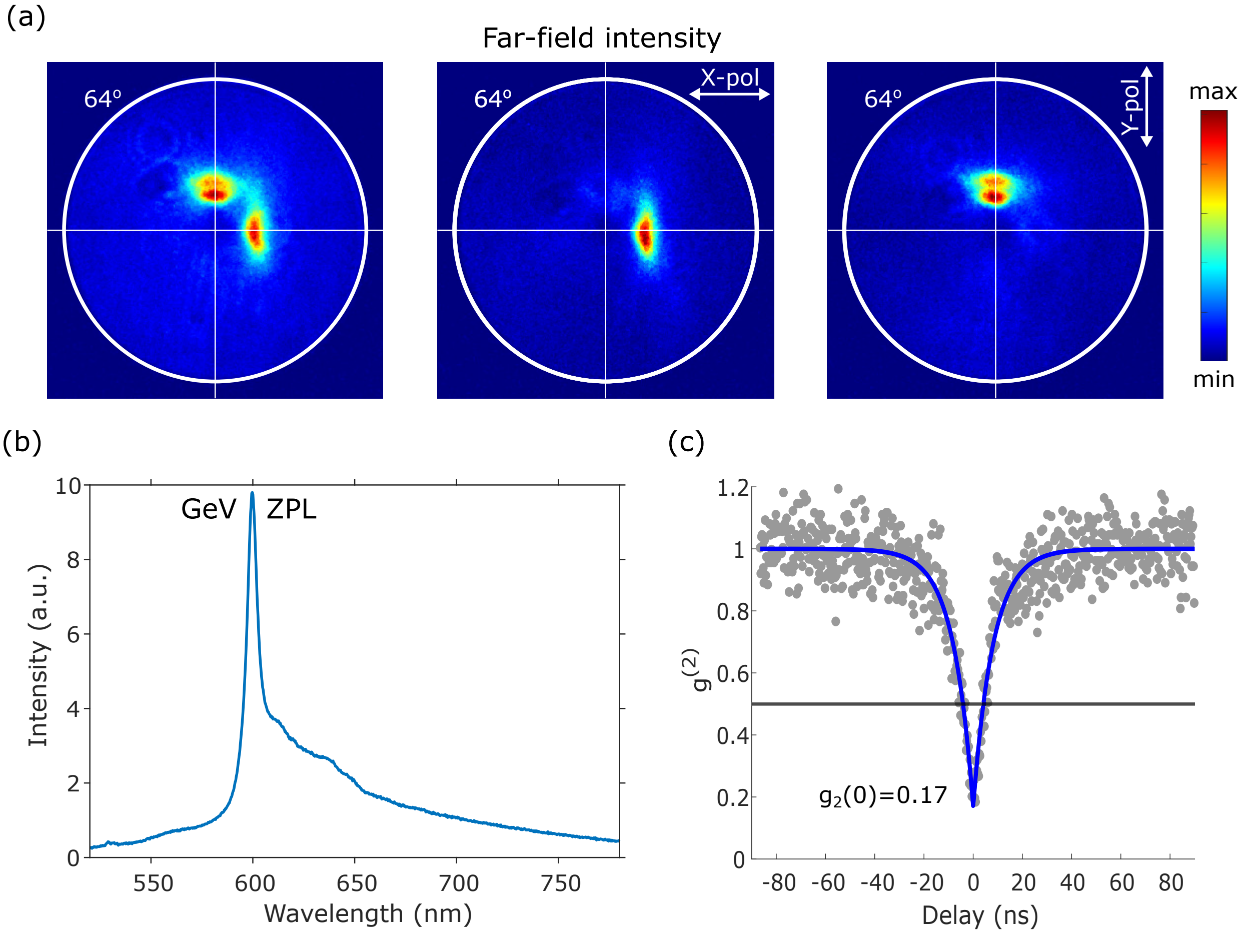

Figure S14: Emission properties of a single-photon GeV coupled to D = 17 μm hologram metasurface. Emsission was collected with 0.9 NA (64°) objective. (a) Far-field patterns of the metasurface emission showing two separated orthogonally polarized emission lobes. (b) Spectrum of GeV fluorescence with ZPL at 602 nm. (c) Histogram of $g^{(2)}$ autocorrelation function.

To prove experimental repeatability, we present emission properties of another single-photon GeV-ND coupled to a different hologram metasurface from the one presented in the main part of the manuscript. Investigation of the far-field emission distribution shows two separated prominent intensity peaks of orthogonal polarization propagating with 15° deflection from the normal to surface presented in Figure S14(a). x-polarized peak has a split shape because of ND

displacement from the central position due to an error in the positioning method. The error in positioning, however, can be reduced by decreasing the distance between reference markers and, therefore, increasing the resolution of positioning. The fluorescence spectrum presented in Figure S14(b) has a clear and sharp peak at 602 nm, which corresponds to the GeV ZPL. The single-photon nature of the exploited ND is examined before metasurface fabrication and proved by the $g^{(2)}(0) = 0.17$ autocorrelation function measurement shown in Figure S14(c). Therefore, we conclude that another hologram metasurface performs the expected function, producing two well-separated collimated beams of orthogonal linear polarizations.

S7.2 Characterization of hologram metasurface coupled to SiV ND

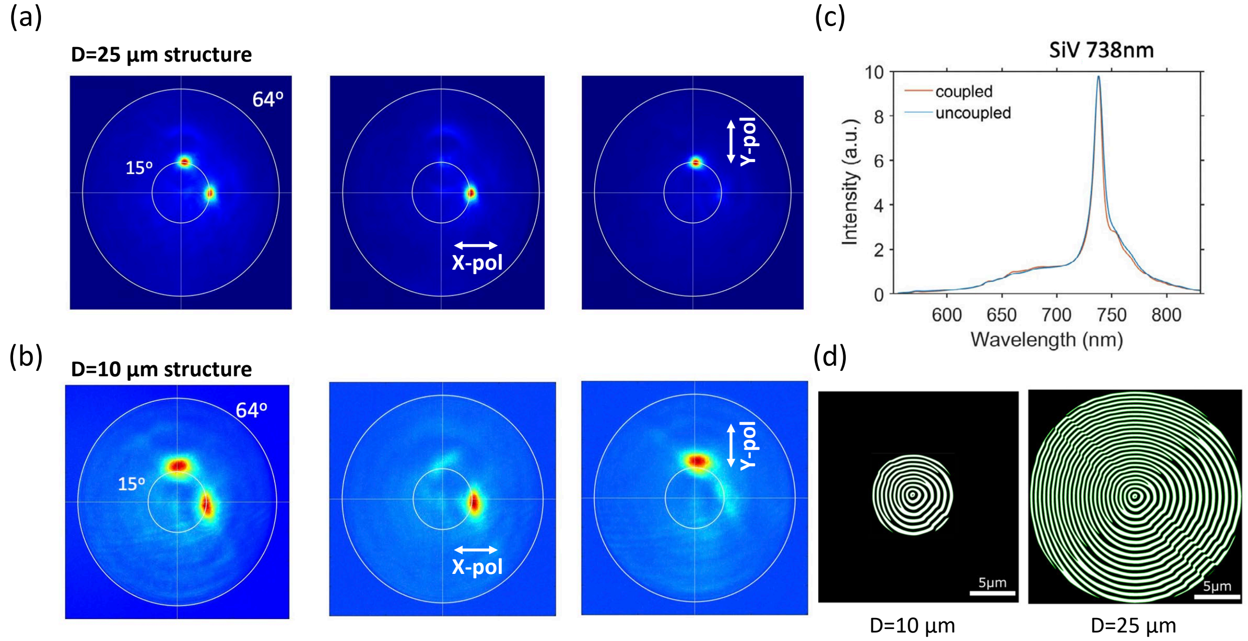

Figure S15: Experiment repeatability demonstration with multiple SiVs in an ND coupled to D = 10 μm and D = 25 μm metasurfaces. 532 nm CW laser was used for excitation. Emission is collected with 0.9 NA objective (collection in 64° cone). (a-b) Measured at the back focal plane far-field patterns of the metasurface emission, representing two separated orthogonally polarized emission lobes for D = 25 μm metasurface (a) and D = 10 μm metasurface (b) . It is clearly shown that beams corresponding to larger metasurface size are significantly better collimated. Patterns were measured using the 740 ± 16 nm band-pass filter. (c) Spectrum of SiV fluorescence with ZPL at 738 nm. (d) Side-by-side comparison of the metasurface patterns and sizes represented at the same scale.

We also prove an experimental reproducibility with another type of quantum emitter - Silicon Vacancy (SiV) centers in nanodiamonds. We demonstrate the operation of two hologram metasurfaces with diameters 10 μm and 25 μm coupled to multiple-vacancy SiV nanodiamonds. Plasmonic metasurfaces are designed for the emission wavelength 738 nm. A comparison of the sizes of devices is presented in Figure S15(d). The prominent peak of fluorescence intensity at 738 nm is evident in the emission from SiV, and is shown in Figure S15(c). Figures S15(a-b) present back focal plane images of the emission generated by 25 μm and 10 μm metasurfaces, respectively. It is seen in the far-field emission maps, that enlarging the structure, and, therefore,

increasing the beam waist results in remarkable enhancement of the beams' collimation. Overall, both hologram plasmonic metasurfaces operate at 738 nm and produce two orthogonally polarized emission channels, propagating at 15° inclination from a sample normal. Moreover, 25 μm metasurface produces significantly better collimated beams than 10 μm structure, which corresponds to the results of performance comparison of metasurface with different sizes provided in section S2.4.

Supplementary References

(S1) Kan, Y.; Bozhevolnyi, S. I. Molding Photon Emission with Hybrid Plasmon-Emitter Coupled Metasurfaces. *Advanced Optical Materials* **2022**, *10*, 2102697.

(S2) Johnson, P. B.; Christy, R. W. Optical Constants of the Noble Metals. *Physical Review B* **1972**, *6*, 4370–4379.

(S3) Palik, E.; Ghosh, G.; Jovanovich, H. B.; (Firm), K.; Lowrie, W. *Handbook of Optical Constants of Solids*; Academic Press handbook series v. 1; Academic Press, 1985.

(S4) Kan, Y.; Andersen, S. K. H.; Ding, F.; Kumar, S.; Zhao, C.; Bozhevolnyi, S. I. Metasurface-Enabled Generation of Circularly Polarized Single Photons. *Advanced Materials* **2020**, *32*, 1907832.

(S5) Komisar, D.; Kumar, S.; Kan, Y.; Wu, C.; Bozhevolnyi, S. I. Generation of Radially Polarized Single Photons with Plasmonic Bullseye Antennas. *ACS Photonics* **2021**, *8*, 2190–2196.

(S6) Lebsir, Y.; Boroviks, S.; Thomaschewski, M.; Bozhevolnyi, S. I.; Zenin, V. A. Ultimate Limit for Optical Losses in Gold, Revealed by Quantitative Near-Field Microscopy. *Nano Letters* **2022**, *22*, 5759–5764.

(S7) Pors, A.; Bozhevolnyi, S. I. Quantum Emitters near Layered Plasmonic Nanostructures: Decay Rate Contributions. *ACS Photonics* **2015**, *2*, 228–236.

(S8) Ford, G.; Weber, W. Electromagnetic interactions of molecules with metal surfaces. *Physics Reports* **1984**, *113*, 195–287.

(S9) Siampour, H.; Kumar, S.; Davydov, V. A.; Kulikova, L. F.; Agafonov, V. N.; Bozhevolnyi, S. I. On-chip excitation of single germanium vacancies in nanodiamonds embedded in plasmonic waveguides. *Light: Science & Applications* **2018**, *7*.

(S10) Wu, C.; Kumar, S.; Kan, Y.; Komisar, D.; Wang, Z.; Bozhevolnyi, S. I.; Ding, F. Room-temperature on-chip orbital angular momentum single-photon sources. *Science Advances* **2022**, *8*.
